# Pseudoaneurysm Accompanied by Crowe Type IV Developmental Dysplasia of the Hip: A Case Report

**DOI:** 10.1155/2012/973489

**Published:** 2012-05-08

**Authors:** Hirotake Yo, Hirotsugu Ohashi, Fumiaki Inori, Yoshiaki Okajima, Yoshio Matsui, Kosuke Shintani

**Affiliations:** Department of Orthopedic Surgery, Osaka Saiseikai Nakatsu Hospital, 2-10-39 Shibata, Kita-ku, Osaka 530-0012, Japan

## Abstract

We report the case of a 72-year-old woman whose pseudoaneurysm was difficult to diagnose and treat. The patient had a history of congenital dislocated hip and was undergoing anticoagulation therapy with warfarin due to the mitral valve replacement. Her chief complaint was pain and enlargement of the left buttock, and the laboratory tests revealed severe anemia. However, her elderly depression confused her chief complaint, and she was transferred to a psychiatric hospital. Two months after the onset of the symptoms, she was finally diagnosed with a pseudoaneurysm by contrast-enhanced CT and angiography. IDC coils were used for embolization. A plain CT showed hemostasis as well as a reduced hematoma at 2 months after the embolization. The possible contributing factors for the pseudoaneurysm included bleeding due to warfarin combined with an intramuscular hematoma accompanied by Crowe type IV developmental dysplasia of the hip that led to an arterial rupture by impingement between pelvis and femoral head. Since the warfarin treatment could not be halted due to the valve replacement, embolization was chosen for her treatment, and the treatment outcome was favorable.

## 1. Introduction

Anticoagulation therapy is used to treat and prevent thromboembolic diseases such as myocardial infarction and cerebral infarction, as well as to prevent catheter occlusion. However, hemorrhagic events are a severe adverse effect of anticoagulant therapy. We report a case of pseudoaneurysm in a patient with Crowe type IV developmental dysplasia of the hip undergoing anticoagulation therapy.

## 2. Case Presentation

The patient was a 72-year-old female with bilateral Crowe type IV developmental dysplasia of the hip. Her chief complaint was enlargement and pain in her left buttock. Her history included congenital dislocated hips and elderly depression. She underwent a valve replacement 2 years prior due to mitral insufficiency and had been taking 1 mg of warfarin and 100 mg of aspirin daily. There was no appreciable family history.

She experienced enlargement and severe pain in the left buttock on April 26 without an obvious etiology such as trauma. The severe pain led to self-injury, and she was transported to an emergency hospital in hemorrhagic shock. She was diagnosed with elderly depression and was transferred to a psychiatric hospital on May 7th. Her laboratory tests showed that she had severe anemia (Hgb 5.1 g/dL). Thus, she was transferred to Hospital A on June 8th and underwent systemic examinations for anemia, including colonofiberscopy. However, a bleeding source such as gastrointestinal hemorrhage was not found. She was transferred to Hospital B on June 19th for further examinations and then came to our hospital on July 1st due to a suspected hematoma in her left hip joint as well as suppurative hip arthritis.

At the time of admission, her height was 148 cm, and her weight was 40 kg. Her blood pressure was 93/58 mmHg with a pulse of 62/min. Her body temperature was 35.9 degrees Celsius. She experienced severe motion pain in her left hip joint with flexion contracture. Her left buttock, groin, and proximal thigh were significantly enlarged, and the skin was tense. The blood laboratory tests revealed high levels of WBC at 10700/*μ*L, and the CRP was 10.8 mg/dL. The severe anemia was reported as follows: RBC 171 × 10^4^/dL, Hb 5.5 g/dL, Ht 18%, and platelets 28.5 × 10^4^/dL, PT-INR 1.34. Ultrasonography expressed a cystic lesion in her left buttock, and echo-guided paracentesis showed a blood-like fluid.

**Figure 1:**
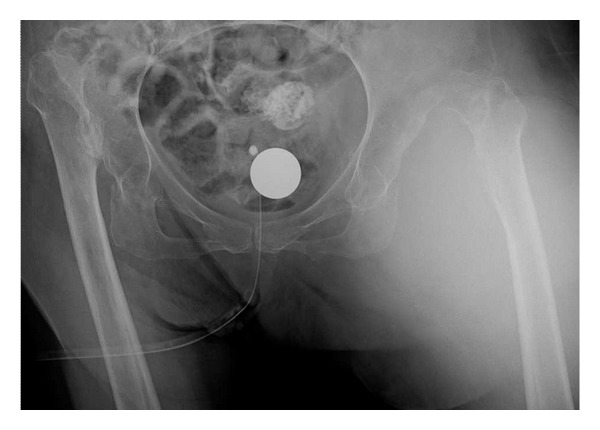
Radiograph taken at admission.

**Figure 2:**
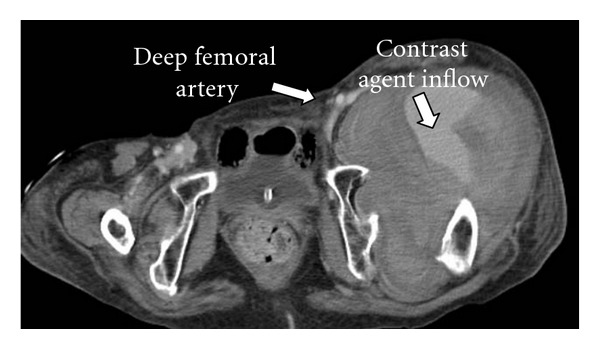
Contrast-enhanced CT.

**Figure 3:**
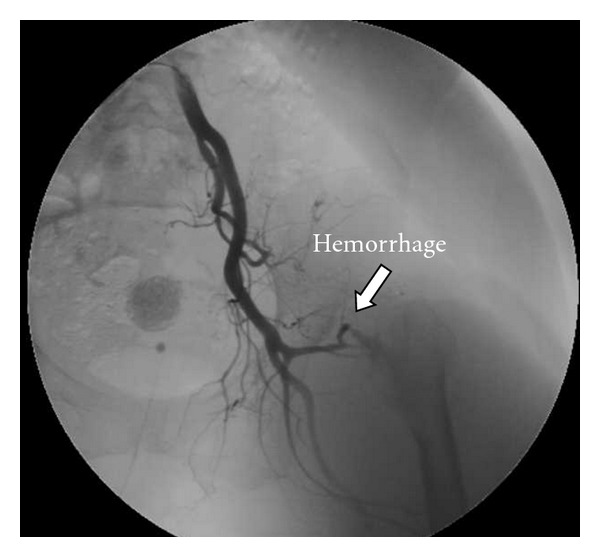
Angiography.

**Figure 4:**
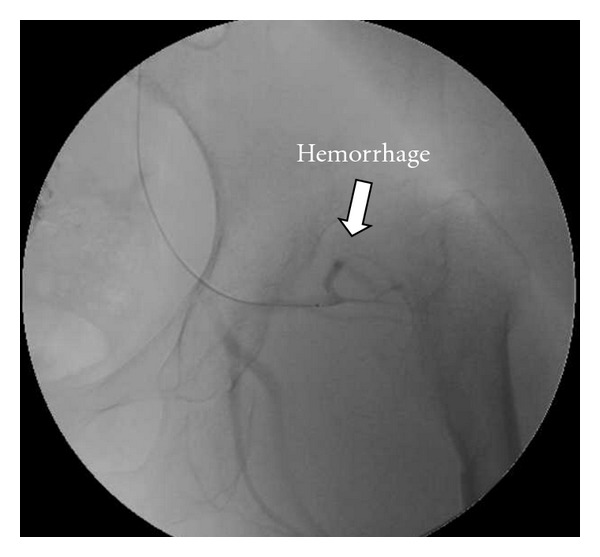
Selective angiography.

Radiograph showed bilateral Crowe type IV developmental dysplasia of the hip (Figure 1). A plain CT showed a cavity that expanded from the left hip joint to the buttock and the thigh. A contrast-enhanced CT revealed extravasation of the contrast medium into the cavity, indicating arterial bleeding (Figure 2). Angiography revealed hemorrhaging from a lateral femoral circumflex artery, and regurgitation from the cavity (Figures 3 and 4). We diagnosed her with a deep femoral artery pseudoaneurysm.

The embolization was chosen for the treatment. A catheter was inserted through the right femoral artery. After a balloon catheter was placed in the left deep femoral arterial circumflex branch to prevent regurgitation, embolization was performed with 3 IDC coils as percutaneous transcatheter angioplasty (PTA).

**Figure 5:**
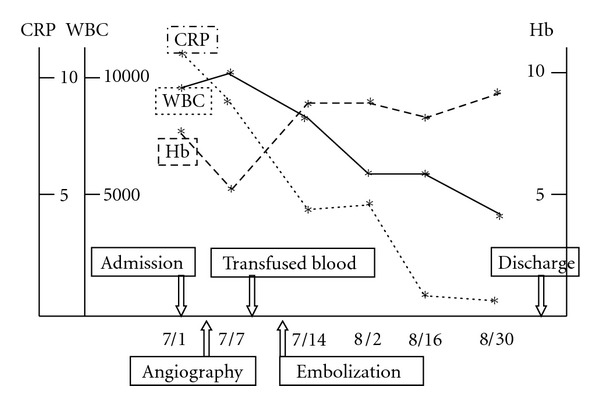
Laboratory results during hospitalization.

**Figure 6:**
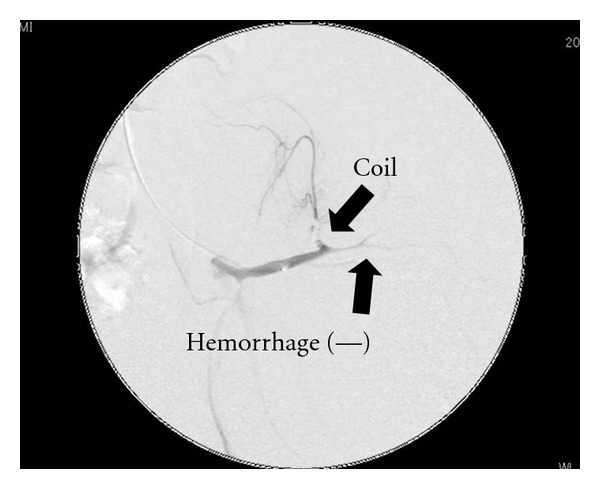
Postembolization angiography.

**Figure 7:**
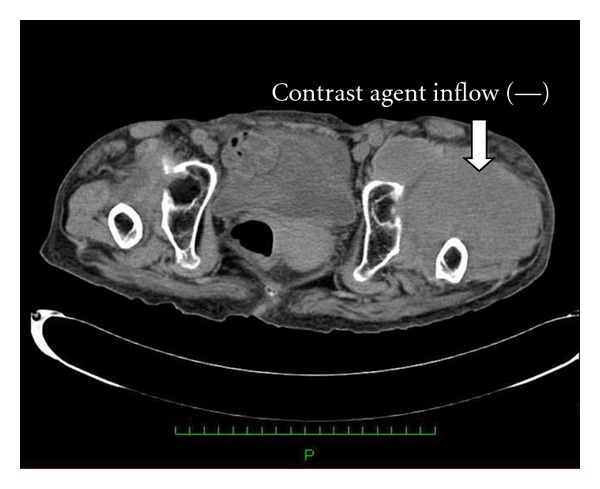
Contrast-enhanced CT scan at 2 months after the embolization.

Her anemia was improved after transfusion and embolization, and the CRP levels dropped to normal levels (Figure 5). Angiography after the embolization showed no signs of hemorrhage or regurgitation from the cavity (Figure 6). A contrast-enhanced CT taken 2 months after the embolization showed no hemorrhage, and the size of the hematoma was reduced (Figure 7). The enlargement and pain in the buttock became mild, and she could walk with a cane at 2 months after embolization.

## 3. Discussion

The characteristics of this patient included anticoagulant treatment, Crowe type IV developmental dysplasia of the hip, severe enlargement and pain in the buttock, severe anemia, and high levels of WBC and CRP. A hematoma, suppurative hip arthritis, a ruptured aneurysm, an infected aneurysm, and deep vein thrombosis, pseudoaneurysm due to arteriovenous malformation (AVM) were considered in the differential diagnosis.

At the beginning, her elderly depression interfered with the diagnosis the hip pain. The hemorrhagic diathesis was suspected from the anticoagulant treatment, thus systemic examinations to find a bleeding source were intended. Since she could not complain her left hip pain because of the elderly depression, it took about 2 months to focus on her left buttock when the enlargement in her buttock was obvious. Contrast-enhanced CT and angiography were useful in making a definite diagnosis [1–3].

Crowe type IV developmental dysplasia of the hip was a possible contributing factor for the pseudoaneurysm in this patient. An intramuscular hematoma within the gluteal muscle would has been easy to occur due to an arterial rupture by impingement between pelvis and femoral head, and the hematoma grew larger because of the hemorrhagic diathesis due to anticoagulant therapy. The infection could have also been another contributing factor, since the ruptured artery would have been caused by inflammation.

The incidence rates of pseudoaneurysm formation at the puncture site in patients undergoing anticoagulation therapy has been reported as 0.05% during catheter examination, and 1.2% during catheter treatment [4]. We could find a few reports of pseudoaneurysms associated with surgical treatment such as THA, and osteosynthesis for femoral neck fractures and femoral intertrochanteric fractures [5, 6]. We could not find a case report of a pseudoaneurysm accompanied by Crowe type IV developmental dysplasia of the hip.

The treatment options for pseudoaneurysm include embolization [3, 7, 8], surgical ligation of the blood vessels combined with hematoma removal [9, 10], and local injection of a coagulating agent. Since warfarin therapy for this patient could not be halted due to the valve replacement, we selected embolization to avoid the risk of bleeding during operation.

There were four reports of hematomas in the iliopsoas muscle in patients who were receiving anticoagulant therapy [9–12]. All of the patients' outcomes were good, with two of them receiving embolization [11, 12] and the other two undergoing surgical ligation of the blood vessels and hematoma removal [9, 10]. Followup was needed for this patient due to the risk factors for rebleeding and infection with hematoma formation because of Crowe type IV developmental dysplasia of the hip and warfarin therapy.

We reported a case of pseudoaneurysm that was difficult to diagnose and treat. Contrast-enhanced CT and angiography were effective in making the diagnosis, and the patient was effectively treated by embolization.
